# Natural sagittal spino-pelvic alignment in boys and girls before, at and after adolescent peak height velocity

**DOI:** 10.1186/1748-7161-10-S1-O15

**Published:** 2015-01-19

**Authors:** Tom P Schlösser, Suken A Shah, Kenneth Rogers, Koen L Vincken, René M Castelein

**Affiliations:** 1 Department of Orthopaedic Surgery, University Medical Center Utrecht, Utrecht, the Netherlands; 2 Department of Orthopaedic Surgery, Nemours/Alfred I. duPont Hospital for Children, Wilmington, Delaware, USA; 3 Image Sciences Institute, University Medical Center Utrecht, Utrecht, the Netherlands

## Objective

One of the unexplained, but well known, characteristics of AIS is that girls are far more often affected than boys and the initiation and progression of the deformity normally occurs around the adolescent growth spurt. From previous studies it can be inferred that, due to biomechanical forces that act on posteriorly inclined spinal segments, certain sagittal spinal profiles are more prone to develop a spinal deformity than others. The aim of this natural history study was to quantify sagittal spino-pelvic alignment and orientation in space of each individual vertebra in normal boys and girls in the beginning, the peak and at the end of pubertal growth.

## Materials and methods

Standardized lateral radiographs of the spine of boys (n=57) and girls (n=99) from 7-18 years old who underwent scoliosis screening and had a normal spine were enrolled. Children with spino-pelvic pathology at initial screening or during follow-up were excluded. According to Dimeglio, subjects were classified into before, at and after the peak growth spurt based on skeletal maturity parameters. Seven regional sagittal spino-pelvic parameters and exact inclination of each individual vertebra between C7 and L5 were measured semi-automatically.

## Results

Thoracic kyphosis, pelvic incidence and pelvic tilt were lower, the posteriorly inclined segment was longer and T1-T8 were more posteriorly inclined before and at the peak of the growth spurt compared to after the growth spurt (P≤0.023) in all subjects. In girls, thoracic kyphosis was smaller (P=0.023), the posteriorly inclined segment was longer (P<0.001) and T1 and T3-T11 were more posteriorly inclined (P<0.05) compared to boys. At the peak of the growth spurt, girls still had a relatively lower thoracic kyphosis with more posterior inclination whereas boys already developed a greater thoracic kyphosis with less posterior inclination (P=0.005).

## Conclusion

These results imply that the spine of girls during the growth spurt is more posteriorly inclined, affected by greater posteriorly directed shear loads and rotationally less stable compared to boys and compared to girls after the growth spurt. This may explain why initiation and progression of AIS frequently occurs in girls around puberty.

